# Assessing clinical support and inter-professional interactions among front-line primary care providers in remote communities in northern Canada: a pilot study

**DOI:** 10.3402/ijch.v75.32159

**Published:** 2016-09-14

**Authors:** Stephanie K. Young, T. Kue Young

**Affiliations:** 1 Institute for Circumpolar Health Research, Yellowknife, Canada; 2 School of Public Health, University of Alberta, Edmonton, Canada

**Keywords:** primary care, northern Canada, remote and rural, nurses, physicians, professional communication, air ambulance

## Abstract

**Background:**

Primary care in remote communities in northern Canada is delivered primarily by nurses who receive clinical support from physicians in regional centres and the patient transportation system. To improve continuity, quality and access to care in remote northern communities, it is important to understand the perspectives of front-line providers and the complex challenges they face.

**Objective:**

To design and implement a survey of primary care providers to identify issues relating to inter-professional communication, clinical support and patient evacuation.

**Methods:**

In collaboration with the territorial government and regional health authority partners, we developed a 21-item self-administered questionnaire survey, which could be completed online. The survey was sent to 218 physicians and nurses who were employed in the Northwest Territories (NWT) at the time of the survey and were involved in sending patients out of the community and/or receiving patients. The survey also contained an open-ended question at the end seeking comments regarding primary health care.

**Results:**

The overall low response rate of 39% among nurses and 19% among physicians threatens the validity of the quantitative results. The majority of providers were satisfied with their ability to communicate with other providers in a timely manner, their freedom to make clinical decisions and their overall experience practicing in the NWT. The patient transfer system appears to work from both the sender and receiver perspectives. However, a common theme reported by nurses was that physicians providing clinical advice, especially short-term locums, were not familiar with the local situation, whilst physicians at the receiving end remarked that the clinical information provided to them often lacked clarity.

**Conclusions:**

Important lessons were learnt from the pilot study, especially in better engagement of providers in planning and dissemination. The questionnaire design and the online method of delivery were acceptable. Although important issues were identified, a larger definitive survey is needed to investigate them in the future.

Primary care is a cornerstone of health care delivery in rural and remote communities in northern Canada. Ensuring quality, accessibility, cultural safety and continuity of primary care remains a pressing priority in northern regions and for the Indigenous populations that live there. In the North, geographical remoteness, low population densities and harsh climates contribute to serious logistical, financial and human resource challenges to delivering care (1,2). Understanding the perspectives of front-line primary care providers is critical to ensuring access to primary care, improving population health and increasing responsiveness to community needs (3).

In the Canadian North, outside the large towns, primary care is provided by nurses stationed in community health centres, supported by physicians who periodically fly into the communities for short visits. When not on site, these regional physicians provide clinical consultations to the nurses using telephone and telehealth systems. When required, patients are transferred (by air) out of the communities – when done on an emergency basis, such transfers are referred to as “medevacs.” This tiered system ensures that basic care is available in all communities whilst more serious cases are transferred for further treatment at a higher level of facility. However, for such a system to function effectively, mutually respectful inter-professional communication, clear guidelines for clinical decision-making and a responsive and timely transportation system are needed. Qualitative research has shown that this is often not the case (4). Problems with clinical support is often cited as a cause of dissatisfaction with working conditions and contributes to poor retention and a high turnover of community-based providers (5–7).

There is an extensive literature on studies conducted in rural/remote settings involving interactions between primary care practitioners and off-site specialists, the use of multidisciplinary teams, telephone triage, centralized dispatch and other forms of support. A study of telephone consultations between rural general practitioners (GPs) and specialists shows that trust is the key element of inter-practitioner communication as it increases understanding and confidence in the reliability of the information exchanged (8). A systematic review of GP–specialist interactions shows that most physical health outcomes remain unchanged, although patient concordance with treatment tends to improve. Physician behaviour also changes with more rational use of resources and diagnostic tests whilst clinical skills also improve (9). A review of continuous and integrated health services in rural/remote areas finds that programs such as managed and integrated care pathways, outreach, shared care and telemedicine may be associated with greater equity in access, more coherence and greater continuity, although not necessarily reduced costs. A well-functioning primary care system, however, is a prerequisite (10).

Of special relevance to the North, given the complex scope of practice of rural and remote nurses working with limited clinical support and few resources, is the literature on nursing decision-making (11–13), decision-making between nurses and doctors (14), and collaborative practice (15,16). Success in primary care collaboration between nurses and doctors entails responsibility and accountability, coordination, communication, co-operation, assertiveness, autonomy and mutual trust and respect (17). Nurses and physicians use differing lenses to interpret and act on knowledge, and it is important to understand how knowledge can be used to improve and increase collaboration between the two professions.

## Study objectives

In this paper, we report on our experience designing and implementing a survey of primary care providers in northern Canada.

The objectives of the survey were to describe providers’ views of the primary care system, with special focus on inter-professional interactions, clinical support of front-line workers and decision-making in patient evacuation.

We decided to pilot test the survey in the Northwest Territories (NWT), and apply the lessons learnt relating to survey format, questionnaire design and subject recruitment for a later, larger survey encompassing the entire Canadian North.

## Study setting

The NWT is bounded by the 60°N latitude to the south and the Arctic Ocean to the North. To its east and west are the two other territories of Canada: Nunavut and Yukon. The NWT is sparsely populated, with a total population of less than 42,000 people, and a population density of 0.04 persons per square kilometre. However, about 46% of the population lives in the capital city of Yellowknife. The NWT is the traditional territory of many First Nations, Inuit and Métis peoples, who collectively account for 52% of the population (2).

Health services are delivered by eight regional health authorities (RHAs), coordinated by the territorial Department of Health and Social Services (DHSS). Vast distances separate the 33 communities in the territory, only a few of which have year-round road access. There are two regional hospitals located in Yellowknife (99 beds) and Inuvik (51 beds) and two smaller ones in Fort Smith and Hay River in the south. In the remote small communities, primary care is delivered through health centres staffed primarily by nurses. In a few communities without resident nurses, community health representatives (CHRs) provide primary care, supported by telephone consultation, and periodic visits by nurses and physicians. In this report, we focused on the perspectives of nurses and physicians. The results of qualitative interviews with CHRs have previously been published in this journal (4). It should be noted that providers are predominantly non-Aboriginal whilst the patients in the communities outside the capital city are predominantly Aboriginal. The NWT has 11 official languages, including nine Aboriginal languages in addition to English and French. The language of health care administration and delivery is most commonly English. Interpreters are used extensively in health care facilities.

In 2015, DHSS revamped and centralized its air ambulance coordination and dispatch system, replacing previous practices whereby different communities had their own channels to specific physicians or directly to a hospital emergency room, with one dedicated number that providers call to secure an air ambulance or external clinical support for urgent and emergent cases (18).

## Methods

### Survey design

We developed a 21-question primary care providers’ survey in collaboration with representatives of the NWT health department and the Nursing Leadership Forum, consisting of the directors of client services in the RHAs. These are individuals who have extensive knowledge of health care delivery and organization and are also the intended end users of the knowledge generated.

The survey was directed at nurses who work in community health centres, primary care physicians who work in regional hospitals and visit the communities, and emergency room physicians who receive patients transferred from the communities. We also sent the survey to eight CHRs (four of whom responded) but they were excluded from the present analysis due to their small number. The present analysis is focused only on nurses and physicians.

The survey included four parts: (a) basic demographic and employment information, (b) providers’ experience seeking and/or providing clinical support and guidance, (c) providers’ experience with the air ambulance system and (d) providers’ overall satisfaction with aspects of their clinical practice in the NWT. The survey consisted of nine multiple-choice questions, five 5-point Likert-scale questions, six questions with short, write-in answers and space at the end of the survey inviting providers to share any additional comments, concerns or suggestions [Question 21]. A copy of the questionnaire can be found in the Supplementary files.

In designing the questionnaire, we were able to adopt three questions verbatim from the Attributes of Primary Health Care: Provider Survey of the Canadian Institute for Health Information (19). These questions [Question no. 18–20 in Part D of the questionnaire] focus on the scope of practice. Whilst the NWT was sampled by both the survey of the Nature of Nursing Practice in Remote and Rural Canada Study (20) and the National Physician Survey (21), they did not inquire about nurse–physician interactions, clinical support or decision-making relating to patient transfer.

### Participant recruitment

Nurses were invited via an email sent from the directors of client services of the RHAs with the approval of the chief executive officers who have access to their employee government email addresses. Physicians were contacted personally by the medical director of the territorial hospital authority. The email provided a link to an online information sheet and consent form. Recipients of the email were given the choice to continue on to the online survey, receive a paper copy in the mail or agree to be approached by the survey team for a telephone interview.

Although the survey was supported by DHSS and the RHAs, we emphasized that this was not a government survey and participation was not a condition of their employment. Participation was voluntary and anonymous and no financial remuneration was offered. The participants were aware that aggregated results would be shared with DHSS and the RHAs.

### Sampling and data collection

The survey was conducted between 1 February and 31 March 2015. Invitational emails were sent to 104 physicians and 114 nurses who were working in the NWT at the time of data collection were surveyed. One reminder follow-up email was sent to all providers midway through the data collection period. The online survey was created using the web-based survey software SurveyMonkey^®^ (22).

### Qualitative data

We decided at the outset to include one open-ended question to enable respondents to expand on their responses [Question 21]. We used thematic analysis for the qualitative data from the open-ended question. Sixteen nurses and seven physicians answered this question, which was intentionally broad in scope. This breadth and the small number of respondents made thematic analysis a suitable method (23). We followed the protocol described by Braun and Clarke (24). An initial set of codes was generated and these were organized into potential themes. Findings were reviewed for agreement and differences. The emerging themes were discussed, agreed upon and refined to generate clear names for each theme (24).

### Ethical review

Ethics approval for this study was granted by the Health Sciences Research Ethics Board of the University of Toronto (Ref. No.30649), with which the principal investigator of the study was previously affiliated. A research license was granted by the Aurora Research Institute, the licensing agency under the NWT Scientists Act, which undertook its own consultation process with participating communities (License No.15564).

## Results

### Response rates

Among eligible participants approached by the invitational email who agreed to participate, all opted for the electronic survey and declined the offer of a mail-in hardcopy questionnaire or telephone interview.

Out of 104 email invitations sent, only 20 physicians responded, which indicates a response rate of 19%. Nurses were more enthusiastic, with 44 responding out of 114 invited, indicating a response rate of 39%.

The low response threatens the validity of the study, but is in itself an important methodological observation about conducting surveys in the remote setting. Moreover, the responses to the open-ended question provided by some respondents were treated as qualitative data and offered important insights.

This paper focuses on nurses who sent patients out of the communities and physicians who receive patients from the communities. The experience of nurses who receive patients from CHRs was not included.

### Demographic and employment characteristics

The majority of respondents (74%) were permanent staff. The range of years spent working in the NWT varied from less than 1 year to 26 years, with an average of 8 years. Most providers were involved with sending patients out of the community (98.5%) and many were also involved with receiving patients from the community (46%).

At least three providers from each health authority participated in the survey. Of note, 31% of respondents worked in one region and 10% of respondents worked in more than one health and social services authority. Most nurses were appointed for short term and many worked several weeks in one region and next time they might be assigned to another region, which could affect their response.

### Inter-professional communication and clinical support

Thirty-one nurses (71%) agreed that the time it took to receive clinical support and guidance was generally acceptable. A similar proportion of nurses (76%) agreed that they had consistent access to clinical support and guidance when required, whilst 27 nurses (61%) agreed that they had immediate access to clinical support when they required it. Only 23 nurses (53%) agreed that the physicians taking their call usually understood the conditions under which they worked.

When recalling the last time they required clinical support and guidance, nurses made on average 2.5 calls and talked to two people. On average, it took them 35 minutes (range: 1–140 minutes) to reach the physician. Over the course of the week, nurses made on average 4.6 calls, lasting 20 minutes.

Physicians who provided clinical support and guidance to nurses working in the communities received on average 10 calls per week, spending on average 20 minutes (range: 5–30 minutes) per call.

The write-in responses offer additional information. Several nurses characterized the physicians they interacted with as having a “general lack of understanding” of the staffing conditions and the scope of care that nurses are able to provide in the communities. Nurses in the community health centres described the challenge of having referrals denied or delayed, and being expected to care for patients “in critical conditions or [those that] require extensive monitoring.” Several nurses reported the lack of adequate staff to care for patients overnight:

There seems to be a general lack of understanding by the ER physicians at [the Regional Hospital] as to the capability of the health centre staff to keep patients for an extended period of time; e.g. I've been asked to hold a patient overnight – we are a 3 nurse station and frequently work short, if I'm held overnight for a patient who would have been appropriate to Medevac there are only 1 or 2 nurses the next day to operate the clinic reducing the clinic's function to emergency services only. [Nurse A]

On the other hand, one nurse acknowledged that “full time MD's that travel to the communities are more likely to understand the conditions community nurses work under. They are more likely to give appropriate advice and ask appropriate questions.” Other write-in responses described physicians working in a regional centre as “very approachable” and “happy to teach.”

A particular issue was the management of patients experiencing mental health challenges. One nurse expressed concerns about the additional time and effort required to obtain adequate care for their patients with mental health concerns:

The most difficult client to find care for is a mental health client. The [territorial health centre] has only 10 beds. If they cannot accept the client into a bed, they will not accept the client at all. There is no middle ground as to performing the psychiatric evaluation. The communities are left with this client in a state of mental collapse, and [the territorial health centre] feels no obligation to assist in finding southern placement for the client. They should not be turning their backs on the small communities with no supports and security, to deal with mental health clients. Our health authority has spent HOURS calling southern hospitals to find somewhere to send these clients. We do not have the resources to be doing this. […] If our staff feel the client needs to be transferred out, that is good enough for me. [Nurse B]

### Responsiveness of the air ambulance system

When reflecting on sending patients out of the communities to another facility, 84% of nurses agreed or strongly agreed that their assessment of the patient's condition and need for evacuation was usually accepted. In contrast, only 53% of nurses agreed that the response time for an air ambulance to be dispatched was generally acceptable, whilst 29% disagreed or strongly disagreed.

When receiving patients from the communities by air ambulance, 80% of physicians agreed that their instructions for clinical management prior to the transfer were usually understood and implemented, and that the pre-transfer and in-transit management was usually appropriate. However, only 33% of physicians agreed that the clinical information provided to them on the phone was clear and relevant, and only 28% considered the clinical information accompanying the patient was clear and succinct, whilst almost half (47%) of the physicians disagreed or strongly disagreed with that statement.

Providers’ assessment of the air ambulance system performance varied. Some nurses felt that the new centralized coordination system for medical transportation and clinical guidance improved physician response time and improved professionalism and courteousness of both parties over the phone because calls were now recorded. There were also opposite views that the response time had not improved; instead, the new system necessitated extra administrative hurdles to reach a referral physician.

The new system has been a bit disappointing and I have found it to be significantly slower and not as user friendly, with more phone calls, more administration time, and a big delay in dispatch of team. This has resulted in some fairly major compromises to patient care. [Physician A]

Other sources of discontent with the air ambulance system included frustration with having to repeat clinical information (i.e. management plans and patient demographics) to multiple providers when requesting an evacuation, whilst they were also “trying to manage the patient and organize transfer documents” [Nurse C].

I also find it time consuming to have a long discussion with the physician about management and then have to repeat everything for the paramedic. It is frustrating. [Nurse D]

Some reported that the expected time of arrival of the air ambulance was poorly communicated.

My concerns about Medivacs are the nurses […] sometimes seem to be the last to be involved in communication – i.e. I don't know … where the plane is, or I am told the medics have left the hospital but then discover they will be sitting at the airport for half an hour while the plane is being warmed up. [Nurse E]

Two physicians expressed concern about the amount of time transport nurses spent providing additional care on the ground which caused a delay in patient evacuation. One physician wrote: “their role as transport team, not diagnosticians, is unclear at times” [Physician B].

To address these gaps in understanding, one nurse suggested that all providers involved with the medical transport team be given an orientation to the different resources available at the community health centres, to “ensure that they take necessary equipment and medications with them, such as oxytocin and equipment needed for preterm delivery in an isolated community that has no nurse on staff at any time” [Nurse F].

### Primary care in the communities

Most providers agreed that the primary care system served the needs of the people of the NWT. In general, nurses reported using more of their full scope of practice in comparison to physicians. All providers reported being very satisfied or somewhat satisfied with several aspects of their primary care practice including the freedom they have to make clinical decisions, their overall experience practicing their profession and their ability to communicate with other providers involved in a timely manner to advance the care of the patient. Most physicians were very satisfied or somewhat satisfied with their ability to remain knowledgeable and current with the latest developments in their field of practice, the time they have available to spend with each patient and the level of understanding others have of their scope of practice. In contrast, just over 20% of nurses reported being not very satisfied or not satisfied at all with those aspects.

Many nurses were not content with the limited amount of time they had to spend with each patient. Due to heavy workloads and understaffing, it was clear that nurses desired more time to spend with each patient. Several nurses also identified the need for additional continuing education and training.

Management likes to assume time-based care and limit same, this subtracts from satisfaction with job and leaves the client being rushed and hurried, which culturally is not normal here. This expectation detracts from client satisfaction too, as time is often the limiting factor and not all things can be properly dealt with in the imposed time frame. [Nurse G]

## Discussion

This study explored the attitudes and experiences of primary health care providers in a remote region of Canada and identified several barriers and facilitators in service delivery. There were challenges related to inter-professional communication, responsiveness of the air ambulance system and workload. Despite these challenges, providers were generally satisfied with practicing their profession in the North, their ability to communicate with other providers in a timely manner and freedom to make clinical decisions. These were aspects that have been identified as positive predictors of provider retention in rural and remote areas (5,6). Although there exists a large literature on inter-professional interactions and clinical decision-making (8–13), especially between nurses and family physicians (14–17), little has been published in the remote, circumpolar context (25).

The low response rate is a cause of concern. As a pilot, the value of this study was in field-testing the acceptability of the questions and the method of distribution. The qualitative data from the write-in responses thus assume more importance, whereas the quantitative results are suggestive rather than definitive, serving to alert us to trends that require confirmation in future studies.

A response rate of 57% – higher than our 39% – was obtained in the NWT and Nunavut portion of a survey of nurses working in remote and rural settings in Canada (20). This mail survey was administered via the provincial and territorial nursing associations and may have contributed to a stronger sense of commitment from the nurses. We intend to repeat the survey in the NWT and include also Nunavut in the near future, in collaboration with the single nursing association that represents both territories. We found that email dissemination of the online survey was feasible, and indeed preferred. The questions used were acceptable and need only minor changes.

We recognized that we had not consulted front-line providers at a very early stage; rather, we relied solely on the input of departmental planning and evaluation staff. In the future, we will engage with front-line providers at an early stage of survey design and dissemination. In post-survey debriefing with the nursing directors, we learnt that some staff members in one particular region – which reported the highest response rate – were resistant to the introduction of the new air ambulance system and may have been motivated to complete the survey to express their dissatisfaction.

Although the response rate of 19% among physicians was very low, it was in fact typical of surveys involving physicians. The response rate among physicians in the 2014 *National Physician Survey* was 16% nationally and 18% in the three northern territories combined (21). That survey was sponsored and commissioned by three national physician organizations and yet still could not generate more than 20% in any province or territory.

A recurrent theme from the responses of nurses was that physicians providers responding to their calls for clinical support or referrals, particularly locum physicians, did not understand the context they were working under. This lack of understanding was a cause of delayed evacuations, resulting in nurses having to carry out prolonged and intensive patient monitoring and providing treatment that were beyond their scope of practice. Physicians who were familiar with the community and its people, and the capabilities of the staff in the local health centres, were regarded as more likely to provide relevant and timely clinical support and guidance. The problem was exacerbated when the providers at both ends of the referral were short-term locums. Improved orientation to the nature of the practice they will experience and organization of health services in the communities should help reduce some of the tensions in inter-professional communication.

When reflecting on the air ambulance system in the NWT, providers emphasized that evacuations varied widely in terms of timeliness. There was a mixed review of the new air ambulance coordination system in improving response time or administrative efficiency. Since the new system was in an early stage of implementation at the time of the survey, it is possible that some areas of concern would have been addressed as providers become more acquainted with the new system. The NWT shares the challenges in other remote air ambulance systems such as limited resources, unpredictable weather conditions and long distances separating suitable landing sites (26). Our findings reveal that accurate information exchange between providers along the trajectory of the evacuation is critical, from the initial phone contact describing the clinical condition of the patient to documenting in-transit care and conveying aeronautical information such as being notified of the estimated time of arrival of the plane.

The perspectives of CHRs, who deliver health promotion interventions and bridge cultural barriers, were not captured due to their small number. However, in a companion interview study, they were included among the key informants (4).

Nurses expressed concern over the difficulty of transferring out patients with mental health problems. Residential facilities to receive such patients were in short supply, even in the territorial capital, whilst the communities had neither the staff nor facilities to hold them or provide appropriate care. The lack of accessible and culturally appropriate mental health services is common across the remote North, and often informal practitioners have to be relied on (27,28).

This study offers useful lessons in the design and conduct of a survey of primary care providers in a remote region, which will be taken into consideration in the planning of a future larger and definitive survey. The qualitative, more so than the quantitative, data offer some insights into the problems (and potential solutions) of sustaining a primary care system that relies on effective communication among providers at different levels in the system and the timely and safe transportation of patients.

## Supplementary Material

Assessing clinical support and inter-professional interactions among front-line primary care providers in remote communities in northern Canada: a pilot study
