# Supplementary material for: Assessing clinical support and inter-professional interactions among front-line primary care providers in remote communities in northern Canada: a pilot study
Source: Int J Circumpolar Health. 2016 Sep 14;75:10.3402/ijch.v75.32159. doi: 10.3402/ijch.v75.32159 (PMC5025523; doi:10.3402/ijch.v75.32159)
Supplement: Assessing clinical support and inter-professional interactions among front-line primary care providers in remote communities in northern Canada: a pilot study [file IJCH-75-32159-s001.pdf]

UNIVERSITY OF TORONTO
DALLA LANA SCHOOL OF PUBLIC HEALTH

Survey of Primary Care Providers in the Northwest Territories

Information Sheet

As a ***health care provider*** in the Northwest Territories, you are invited to participate in a survey conducted by the Circumpolar Health Systems Innovations Research Team (CircHSIT) in collaboration with the NWT Department of Health and Social Services and regional health authorities.

CircHSIT is a 5-year research partnership involving university researchers and decision makers in territorial and regional health agencies focused on strengthening and improving the health system in the North. It is funded by the Canadian Institutes for Health Research and led by Dr. Kue Young of the Dalla Lana School of Public Health, University of Toronto.

The purpose of the survey is to solicit the views and opinions of health services providers in the NWT regarding the patient transportation system (part B), clinical support and guidance (part C) and primary health care more broadly in the communities (part D). Such a survey is timely as a new coordinated medical travel and emergency evacuation system known as Med-Response is in its early stage of implementation and due to be fully launched early in 2015.

The survey is directed at three types of providers in the NWT:

1) Community Health Representatives working in communities without nurses;
2) Community health nurses in the community health centres, including nurses-in-charge;
3) Primary care physicians in regional hospitals who visit the communities and care for patients from those communities and emergency room physicians who receive patients transferred from the communities;

The survey consists of four parts:

A) General information about you
B) Your experience with the air ambulance system
C) Your experience seeking and/or providing clinical support and guidance
D) Your views about primary health care provided at the community level

The survey is entirely anonymous, confidential and voluntary. **You may choose not to answer any question and withdraw at any time. The survey will require about 30 minutes of your time.** There are three options: (1) We can send the link to the online survey by email and you can complete it online; (2) we can mail you a hardcopy questionnaire to be filled out and returned to us by mail; or (3) we can set up a suitable time and conduct the survey over the telephone. There will be no face-to-face interview/visit by the research team.

Although the senior leadership of the NWT Department of Health and Social Services and the regional health authorities were consulted in the design of the survey, this is a research project and not a requirement of your job. Respondents cannot be identified individually and individual level responses are not available to the government of the NWT. How you respond to the questions will not affect your performance evaluation and have no negative legal or administrative consequences. There is no physical, psychological or social harm in participating in the survey. You may be inconvenienced, and finding time to participate in the survey during working hours may be difficult given your heavy daily workload.

While the survey has no direct benefit to you individually, the knowledge gained from the survey will be important to health care policy makers and program planners in the NWT in improving primary health care in the communities, especially the medical transportation system. There is no monetary compensation to you for participating in the survey.

Information collected from the survey will be processed and stored at the secure research data centre at the Institute for Circumpolar Health Research in Yellowknife. The dataset will be accessible only to the research team for analysis and report preparation. Results of the survey will be shared with our decision-maker partners in

NWT Health and Social Services and the regional health authorities. A copy of the dataset will be provided to them. No personal identifiable information will be contained in the dataset.

The research team will present the results of the study at scientific and professional conferences or workshops and submitted for publication in health sciences journals.

Please direct any question regarding the study to:
• Dr. Kue Young, 587-335-1155 or email kue.young@utoronto.ca or
• Ms. Stephanie Young, 867-873-9337 or email stephanie.young@ichr.ca

If you are concerned about your right as a study participant, you can contact the University of Toronto Office of Research Ethics at ethics.review@utoronto.ca or 416-946-3273

Thank you for your consideration.

Sincerely,

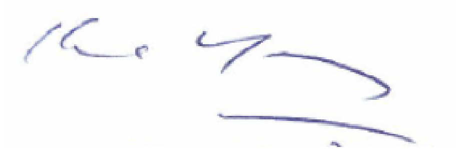

Kue Young, MD, FRCPC, DPhil, FCAHS
Professor Emeritus
Dalla Lana School of Public Health
University of Toronto

Consent to Participate in Survey

Question #1: <u>Please check either Yes or No</u>:

	Yes	No
I understand I have been asked to participate in the *Survey of Primary Care Providers in the Northwest Territories*	☐	☐
I have read the information sheet above	☐	☐
I understand the risks that I may face in taking part in this survey	☐	☐
I understand the benefits of participating in this survey	☐	☐
I understand that my responses to the questions in the survey will be confidential	☐	☐
I have been offered a chance to ask the researchers questions	☐	☐
I would like to receive a summary of the survey results	☐	☐

Question #2: <u>I agree to participate in the *Survey of Primary Care Providers in the Northwest Territories*</u>:

☐ Yes

☐ No

Part A: Respondent Information

Question #3: <u>Which Health Authority do you work in</u>? Check all that apply.

☐ Beaufort Delta Health and Social Services Authority
☐ Dehcho Health and Social Services Authority
☐ Fort Smith Health and Social Services Authority
☐ Hay River Health and Social Services Authority
☐ Sahtu Health and Social Services Authority
☐ Stanton Territorial Health Authority
☐ Tłicho Community Services Agency
☐ Yellowknife Health and Social Services Authority

Question #4: <u>What is your primary profession/occupation</u>?

☐ Community Health Representative
☐ Nurse
☐ Nurse in Charge
☐ Nurse Practitioner
☐ Physician
☐ Other (please specify):

____

Question #5: <u>What is the nature of your employment</u>?

☐ Permanent staff
☐ Locum/Short Term Contract Staff
☐ Medical resident/ Trainee
☐ Other (please specify):

____

Question #6: <u>How many years (total) have you worked in health care in the Northwest Territories</u>?

☐ Less than 1
☐ More than 1 year (please state the number of years):

____

Part B: Air Ambulance Service

Question #7: <u>Select the option(s) that best describes your role in patient transportation</u>. Check all that apply:

☐ I am involved with **sending** patients out of the community to another facility.

☐ I am involved with **receiving** patients from the communities.

Question #8: <u>Evaluate the following statements about *sending* patients out of the community to another facility</u>. Check N/A if you are not involved with sending patients out of the community:

	Strongly Disagree	Disagree	Neither Disagree Nor Agree	Agree	Strongly Agree	N/A
The response time (between placing the first call to when the air ambulance is dispatched) is generally acceptable	☐	☐	☐	☐	☐	☐
I am usually able to have immediate access to clinical advice on patient management	☐	☐	☐	☐	☐	☐
The response to the patient's emergency health situation is appropriate to the patient's level of acuity	☐	☐	☐	☐	☐	☐
The health care practitioner taking my call usually understands the conditions under which I work	☐	☐	☐	☐	☐	☐
My assessment of the patient's condition and need for evacuation is usually accepted	☐	☐	☐	☐	☐	☐

Question #9: <u>On average, how long (in minutes) does it take from your first call to receiving notice that air ambulance is dispatched</u>? Please leave blank if it is not applicable:

____

Question #10: <u>Evaluate the following statements about *receiving* patients from the communities</u>. Check N/A if you are not involved with receiving patients from the communities:

	Strongly Disagree	Disagree	Neither Disagree Nor Agree	Agree	Strongly Agree	N/A
The clinical information provided to me on the phone is usually clear and relevant	☐	☐	☐	☐	☐	☐
The clinical information accompanying the patient is usually clear and succinct	☐	☐	☐	☐	☐	☐
My instructions for clinical management prior to transfer are usually understood and implemented	☐	☐	☐	☐	☐	☐
The patient pre-transfer and in-transit management is usually appropriate	☐	☐	☐	☐	☐	☐
Patients rarely need to be transferred to a higher-level care facility once you have assessed them	☐	☐	☐	☐	☐	☐

Part C: Clinical Support and Guidance

Question #11: <u>If you work in a community and regularly *make calls* to get clinical support and/or guidance, evaluate the following statements</u>. Check N/A if you do not make such calls:

	Strongly Disagree	Disagree	Neither Disagree Nor Agree	Agree	Strongly Agree	N/A
The response time (time between placing first call to receiving clinical support and/or guidance) is generally acceptable	☐	☐	☐	☐	☐	☐
I have **consistent** access to clinical support and/or guidance by phone when I require it	☐	☐	☐	☐	☐	☐
I have **immediate** access to clinical support and/or guidance when I require it	☐	☐	☐	☐	☐	☐
I receive the necessary support and/or guidance to provide appropriate patient care	☐	☐	☐	☐	☐	☐
The health care practitioner taking my call usually understands the conditions under which I work	☐	☐	☐	☐	☐	☐

Question #12: <u>When thinking about the last time you needed assistance:</u>

How **many calls** did you have to make to obtain the clinical support and/or guidance required? Leave blank if it is not applicable:

____

How **many individual people** did you have to talk to in order to obtain the clinical support and/or guidance required? Leave blank if it is not applicable:

____

How **long did it take from your first call** (in minutes) in order to obtain the clinical support and/or guidance required? Leave blank if it is not applicable:

____

Question #13: <u>On average, how much time (in minutes) does each call take to obtain clinical support and/or guidance</u>? Leave blank if it is not applicable:

____

Question #14: On average, how many calls do you make per week to obtain clinical support and/or guidance? Leave blank if it is not applicable:

____

If you are involved in ***receiving calls*** from a community health representative (CHR) in a community or nurse in a community health centre, answer the following questions. Leave blank if you do not receive calls.

Question #15: On average, how many calls do you receive per week from communities to provide clinical support and/or guidance? Leave blank if it is not applicable:

____

Question #16: On average, how much time (in minutes) does each call take to provide clinical support and/or guidance? Leave blank if it is not applicable:

____

Part D: Primary Health Care in the Communities

Note: Items with an asterisk (*) are taken verbatim from the *Attributes of Primary Health Care: Provider Survey* of the Canadian Institute for Health Information

Question #17: Evaluate the following statements regarding the existing primary health care system in the Northwest Territories:

	Strongly Disagree	Disagree	Neither Disagree Nor Agree	Agree	Strongly Agree	N/A
Overall, this primary health system serves the needs of people of the Northwest Territories	☐	☐	☐	☐	☐	☐

***Question #18: How much of your scope of practice (that is, the complete set of skills you are trained to use) do you actually use over the course of a year?**

☐ I use little of my full scope of practice

☐ I use about half of my full scope of practice

☐ I use most of my full scope of practice

☐ I use my full scope of practice

Question #19: Indicate your level of satisfaction with the following aspects of your primary care practice:

	Not at all satisfied	Not very satisfied	Neutral	Somewhat satisfied	Very satisfied
*My ability to remain knowledgeable and current with the latest developments in my field of practice	☐	☐	☐	☐	☐
*The freedom I have to make clinical decisions that meet my patients' needs	☐	☐	☐	☐	☐
*The time I have available to spend with each patient	☐	☐	☐	☐	☐
*Overall experience with practicing my profession	☐	☐	☐	☐	☐
*The level of understanding others have of my scope of practice	☐	☐	☐	☐	☐

***Question #20: Are you able to communicate with other providers involved in a timely manner to advance the care of the patient?**

☐ Not at all

☐ No, not really

☐ Undecided

☐ Yes, to some extent

☐ Yes, to a very great extent

Additional Feedback

Question #21: **If you have any additional questions, comments, concerns and/or suggestions please feel free to write them here.**

End of Survey

Thank you for completing our brief survey and submitting your valuable feedback. Your confidential responses will help inform and improve primary health care in the communities, especially related to the medical transportation system.
